# Mutation of Proteolipid Protein 1 Gene: From Severe Hypomyelinating Leukodystrophy to Inherited Spastic Paraplegia

**DOI:** 10.3390/biomedicines10071709

**Published:** 2022-07-15

**Authors:** Guy Khalaf, Claudia Mattern, Mélina Begou, Odile Boespflug-Tanguy, Charbel Massaad, Liliane Massaad-Massade

**Affiliations:** 1 U1195 Diseases and Hormones of the Nervous System, INSERM and Université Paris-Saclay, 94276 Le Kremlin-Bicêtre, France; guy.khalaf@inserm.fr; 2 M&P Pharma, Schynweg 7, 6376 Emmetten, Switzerland; info@mattern-pharma.com; 3 Neuro-Dol, CNRS, Inserm, Université Clermont Auvergne, 63000 Clermont-Ferrand, France; melina.begou@uca.fr; 4 UMR 1141, INSERM, NeuroDiderot Université Paris Cité and APH-P, Neuropédiatrie, French Reference Center for Leukodystrophies, LEUKOFRANCE, Hôpital Robert Debré, 75019 Paris, France; odile.boespflug-tanguy@aphp.fr; 5 UMRS 1124, INSERM, Université Paris Cité, 75006 Paris, France

**Keywords:** Pelizaeus-Merzbacher disease (PMD), spastic paraplegia (SPG2), diagnosis, proteolipid protein 1 variants, animal models, treatments

## Abstract

Pelizaeus–Merzbacher Disease (PMD) is an inherited leukodystrophy affecting the central nervous system (CNS)—a rare disorder that especially concerns males. Its estimated prevalence is 1.45–1.9 per 100,000 individuals in the general population. Patients affected by PMD exhibit a drastic reduction or absence of myelin sheaths in the white matter areas of the CNS. The Proteolipid Protein 1 (*PLP1*) gene encodes a transmembrane proteolipid protein. PLP1 is the major protein of myelin, and it plays a key role in the compaction, stabilization, and maintenance of myelin sheaths. Its function is predominant in oligodendrocyte development and axonal survival. Mutations in the *PLP1* gene cause the development of a wide continuum spectrum of leukopathies from the most severe form of PMD for whom patients exhibit severe CNS hypomyelination to the relatively mild late-onset type 2 spastic paraplegia, leading to the concept of PLP1-related disorders. The genetic diversity and the biochemical complexity, along with other aspects of PMD, are discussed to reveal the obstacles that hinder the development of treatments. This review aims to provide a clinical and mechanistic overview of this spectrum of rare diseases.

## 1. Introduction

In the central nervous system (CNS), myelin is produced by oligodendrocytes. The primary roles of oligodendrocytes are axonal ensheathment and myelin formation. Oligodendrocytes wrap their cellular membranes around the axons to form compact myelin in the CNS white matter [1]. Despite its major structural and physical roles, myelin is not a static entity. A cross-talk remains between the oligodendrocyte cell body and myelin [2] as well as between the axon and myelin [3]. Thus, any disruption or dysregulation of the oligodendrocytes will have repercussions on the myelin and subsequently on the axons and the entire white matter [4].

Leukodystrophies (*leuko*, white, and *dystrophy*, degeneration) are a heterogeneous group of inherited disorders characterized by abnormal white matter in CNS [5]. They usually result in progressive neurodegeneration and a premature death [6]. Friedrich Pelizaeus in 1885 [7] described a large pedigree affected by an X-linked form of early–onset slowly progressive motor disabilities. The neuropathological pattern was reported in 1910 by Merzbacher as a white matter disorder suggesting a defective myelination [8]. Therefore, Pelizaeus–Merzbacher Disease (PMD) became the prototype of inborn errors of CNS myelin formation (hypo/dys-myelinating leukodystrophies) in opposition to genetic diseases leading to myelin destruction (demyelinating leukodystrophies). A major advancement in the genetic understanding of the disease was made with the assignment of the PLP1 gene to the X chromosome [9] and the identification of *Plp1* abnormalities in various hypomyelinated animal mutants [10,11]. The introduction of neuro electrophysiological techniques (brainstem auditory and somatosensory evoked potentials) and magnetic resonance imaging (MRI) to demonstrate the abnormal formation and functioning of CNS myelin defined new PMD diagnostic criteria that paved the way for molecular studies [12]. Several point mutations in the coding and non-coding regions of the PLP1 gene [11] have been subsequently associated with the PMD phenotype [13]. The most frequent genetic alteration underlying PMD sporadic forms is the duplication of the entire *PLP1* gene [14] Together, PMD and the X-linked form of spastic paraplegia (SPG2) are allelic diseases related to the *PLP1* locus [15,16], thus increasing the phenotype of PLP1-related disorders. The large number of *PLP1* genetic alterations that disrupt the myelination process have been classified into three main groups: *PLP1* duplications of different sizes affecting 60% to 70% of patients [17,18], the intragenic sequence variants affecting 5% to 20% of patients [18,19], and the complete deletion of the PLP1 gene affecting 2% of patients [20,21].

PLP1-related diseases, and particularly PMD, represent a challenging heterogeneous group of disorders that deteriorate patient quality of life. To date, no effective cure is established, and patients only receive palliative treatments. The genetic diversity and the biochemical complexity, along with other aspects of PMD, are discussed to reveal the obstacles that hinder the development of treatments. This review aims to provide a clinical, mechanistic, and therapeutic overview of this rare disease.

## 2. Clinical Presentation of PLP1-Related Disorders

PMD, also defined as hypomyelinating leukodystrophy type 1 (HLD1), is the most frequent leukodystrophy. Its estimated prevalence is between 1.45 and 1.9 per 100,000 live male births [22,23]. Male patients affected by PLP1 disorders express a large clinical spectrum ranging from the most severe PMD forms (also named “connatal form”) (PMD; OMIM 312,080) to the mildest SPG2 forms (SPG2; OMIM 312,920). The main difference between SPG2 and PMD is in the motor milestone achievement which is almost normal in SPG2 patients.

In male patients, PMD is characterized by an early impairment of motor development (before 6 months of life) paralleled with neurological signs that are gradually modified by the maturing nervous system [24]. Nystagmus, which is present in 93% of cases, increases in intensity during the first months of life and then decreases after the first year with persistent abnormalities in eye coordination and poor visual acuity. Choreoathetotic movements of the limbs and/or ataxia of the head and trunk usually occur after 6 months, decreasing in intensity between 3 and 5 years of age, when progressive spasticity frequently associated with dystonia is observed. This spasticity, related to progressive axonopathy, increases in intensity during teenage years, leading to severe orthopedic deformities (permanent foot deformities, hip dislocation, and scoliosis). Motor handicap is always greater than the impairment of psycho-intellectual development with good social interactions. A decrease in growth velocity (first in weight and subsequently in height) is usually observed between 3 and 5 years of age. Seizures are rarely observed and are well controlled by antiepileptic drugs. No rapid degradation is observed, and the motor performance of most patients improves during the first 5 years of life. After reaching a plateau, a clear deterioration is observed after adolescence with increasing spasticity/dystonia, progressive optic atrophy, swallowing difficulties, and sphincter dysfunctions.

**Figure 1:**
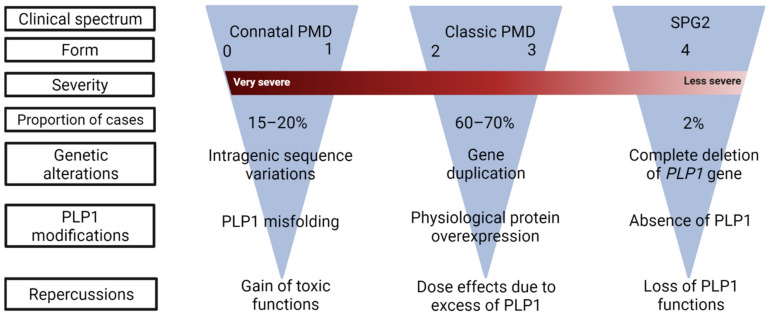
Clinical classification of PLP1-related disorders. The spectrum of the disease is a continuum ranging from severe PMD (form 0) to SPG2 (form 4), with decreasing clinical severity. Under each category, the most frequent *PLP1* mutations and their consequences are indicated.

As for patients with acquired forms of neurodevelopmental white matter disorders (white matter disorder of the premature, cerebral palsy), the severity of the disease correlates with the level of motor performance acquired before 5 years. PLP1-related diseases are classified into five motor developmental scores (MDS): 0, no achievement; 1, head control; 2, sitting position; 3, walk with aid; 4, autonomous walking [24,25] (Figure 1).

Form 0 (10% of cases) has also been individualized as the “connatal” or Seitelberger form. Patients achieve almost no motor milestones with neonatal stridor, early severe ataxia, dystonia, and swallowing difficulties. Severe growth impairment is observed after 1 year of age, despite feeding enteral nutrition. Death usually occurs during the first decade. Form 1 (10%) includes patients who achieve head control at a mean age of one year; one third are able to use words. Severe spasticity, swallowing difficulties, and bladder dysfunctions induce morbidity during the third decade of life. Patients with form 2 (40%) achieve a sitting position around 2 years of age, and half of them have comprehensible but dysarthric speech between 2 and 5 years. In form 3, (30%) patients can walk with some support at the mean age of 2.5 years, but 25% lose this capacity at 10 years of age and all lose it after 15 years of age. The large majority develop an intelligible but dysarthric language at a normal age range and attend a regular school using a personal computer, at least for the first grades. Frontal lobe dysfunctions with poor working capacities impair higher levels of education. Increasing severity of the handicap is observed for both form 2 and 3—also named “classic PMD”. However, survival is not deeply affected (a long survival up to the fifth or even seventh decade is noticed).

**Figure 2:**
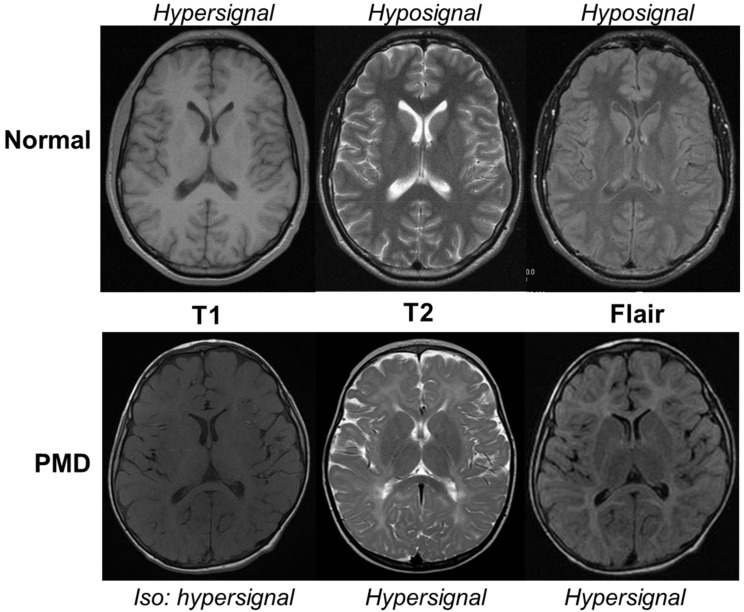
MRI of a 5-year-old Pelizaeus–Merzbacher Disease (PMD) patient in comparison with an age-matched normal boy (Normal). In physiological conditions, the myelinated white matter has a hypersignal T1, hyposignal T2, and flair when compared to gray matter. In PMD, the hypomyelinated white matter has a normal hypersignal T1 appearance contrasting with an abnormal hypersignal T2 and flair. The corpus callosum is partially myelinated with a hyposignal T2 but not with flair.

In all cases, the defect in CNS myelin formation is demonstrated by both dramatic and extensive abnormalities of multimodal evoked potentials in the CNS present since birth that remain stable and by the diffuse hypomyelinated pattern of the sustentorial white matter on MRI. MRI allows an assessment of the state of maturation of the child’s brain [26]. Changes in MRI parameters (shortening of T1 and T2 relaxation times, reduced water diffusion, increased diffusion anisotropy, and increased magnetization transfer) are largely thought to be the result of myelination. As the white matter forms, it changes first from hypointense to hyperintense relative to gray matter on T1-weighted images and then from hyperintense to hypointense relative to gray matter on T2-weighted images. In PMD patients where myelin maturation fails, a diffuse hypersignal of the supratentorial white matter on T2-weighted and fluid-attenuated inversion recovery (FLAIR) sequences contrasting with a normal hypersignal or isosignal on T1-weighted sequences in comparison with grey matter are observed (Figure 2). However, the myelination score increases with time until adolescence, particularly in the early myelinated areas (cerebellum, brainstem, internal capsule, and corpus callosum) in forms 0–1 or in the U fibers and frontal lobes in forms 3–4. Most patients show worsening atrophy (brain, cerebellum, corpus callosum), whereas grey matter and white matter proportions do not change. Brain atrophy and myelination of anterior cerebral regions appear to be pertinent biomarkers of motor development [25].

SPG2 is more rarely observed (10% of cases). In form 4 of PLP1-related disorder, the motor milestones of the first year of life are not impaired, but affected boys develop a progressive ataxia-spastic gait in the following five years. Severe spasticity and loss of ambulation are observed during adolescence. Low-performance IQ and frontal lobe dysfunctions impair a high level of education. Optic atrophy, sphincter dysfunction (spastic urinary bladder), ataxia, or dystonia are present at the end of the second to fourth decade but are compatible with normal survival [27]. A progressive multifocal, predominantly axonal, peripheral neuropathy is frequently observed in SPG2 or mild forms of PLP1-related disorders [28]. Abnormalities in both white matter signals on cerebral MRI and somatosensory/motor-evoked potentials suggest that brain hypomyelination is always observed, even in milder cases.

SPG2-like patients in which hypomyelination is specifically pronounced in early myelinating structures have been reported as a new entity: “hypomyelination in early myelinating structures” (HEMS) and have been related to PLP-specific mutations [29].

PMD-affected females have been rarely reported. In comparison with male patients, improvement is clearly observed after 5 years of age [30]. Injury to the oligodendrocytes during the perinatal period (prematurity, anoxia, or infection), seems to alter the disease expression more than the skewed pattern of X inactivation. Moreover, heterozygous mothers having PLP1 mutations are usually asymptomatic, but their sons exhibit severe phenotypes, while females carrying nonsense/indel or null mutations tend to be symptomatic in the mildest forms of PLP-related disorders [31]. These female carriers express a late-onset form of axonopathy with spastic paraplegia during the fourth decade, often associated with progressive frontal dementia during the sixth decade. In most cases, progressive brain atrophy without white matter abnormalities on MRI is associated with abnormal central and peripheral conductions.

## 3. Molecular Investigations

An increasing number of disease-causing genes are reported in patients with a PMD/SPG2-like phenotype [32]. Therefore, to confirm the PMD/SPG2 diagnosis, molecular confirmation is required.

In PMD, large duplication including the entire *PLP1* gene is the most frequent causative mutation (64%), mainly observed in forms 2 and 3. Triplication seems to be correlated with more severe forms similar to what is observed in transgenic mice overexpressing *Plp1* [14,33,34]. The reference molecular method usually used is multiplex ligation-dependent probe amplification (MLPA). However, chromosomal microarray testing is routinely employed to detect the large majority of duplications.

In sharp contrast, large deletions of the *PLP1* genomic region and null mutations seem to be well-tolerated, allowing for myelination and accounting for the mildest form of PMD or SPG2 [17,18,19,35].

In PLP1 point mutations, genotype–phenotype correlation is less clear [24]. Substitutions of conserved amino acids and truncated proteins with large changes in C-terminal structure have the largest effect on the intracellular interactions of mutated proteins. Mutations in non-coding regions of the *PLP1* gene are missed by exome analysis [33]. Mutations of the intronic enhancers of the *PLP1* splice site have been reported in SPG2/HEMS patients, demonstrating the critical role of *PLP1* alternative splicing regulation in the myelination process [34].

## 4. Proteolipid Protein 1: Substantial Physiological Functions

The proteolipid protein 1 was discovered 70 years ago [36]. It is the most abundant protein of the CNS myelin, accounting for more than 50% of the total protein weight [37]. It is a 276-amino-acid-long polypeptide chain [38] encoded by a single 17-kilobase gene located on the X chromosome and composed of seven exons and six introns [39]. An internal splice donor site [40] is present on the third exon. The isoform of PLP1 obtained by alternative splicing is the DM20 [38,41]. DM20 is a 241-amino-acid protein, and it differs from PLP1 by a deletion of 35 amino acids (116–150) from the major hydrophilic domain [42]. The exon in question is the last to be cleaved, and this process is cell specific and regulated developmentally [43].

PLP1 expression is mainly detected in oligodendrocytes, while myelinating cells of the peripheral nervous system (Schwann cells) mostly express the DM20 isoform [19,44]. Developmental regulations also play a role in the splicing of the pre-PLP1 mRNA [45,46]. During the embryonic development of the CNS, DM20 is predominantly expressed. Then, when the myelinating oligodendrocytes mature, PLP1 takes over to reach a ratio of 3:1 with DM20 [47].

PLP1 is a tetraspan transmembrane 30 kDa protein with NH_2_ and COOH termini in the cytoplasm. It has four transmembrane α-helices inside the membrane of oligodendrocytes, and it is a highly hydrophobic protein. In addition, fatty acids found in the membrane also interact with PLP1. Palmitic acid is covalently bound to PLP1 via six cysteine residues [47] by an autocatalytic posttranslational modification [48]. The fatty acids attached to the intracellular loop of PLP1 ensure the integration of this protein in the lipid leaflet in compact myelin [47].

**Figure 3:**
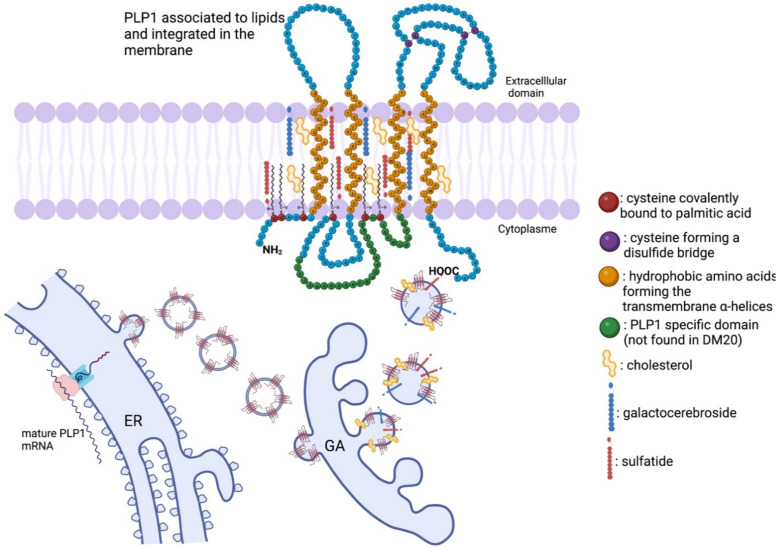
The physiological state of PLP1 formation: from mRNA to a functional transmembrane protein. The mature mRNA encoding PLP1 is translated in the rough endoplasmic reticulum (ER). Afterward, the immature protein is transported to the Golgi apparatus (GA), where it will associate with lipidic myelin components, such as cholesterol, galactocerebrosides, and sulfatides to form lipidic rafts. Then, the lipidic rafts are sent toward the cellular membrane to form the myelin sheath. PLP1 is a 30 kDa tetraspan transmembrane protein with NH_2_ and COOH termini in the cytoplasm. It has four transmembrane α-helices inside the membrane of oligodendrocytes, and it is a highly hydrophobic protein. Palmitic acid is covalently bound to PLP1 via six cysteine residues by an autocatalytic posttranslational modification. The fatty acids attached to the intracellular loop of PLP1 ensure the integration of this protein in the lipid leaflet in compact myelin.

The mature mRNA encoding PLP1 is translated in the rough endoplasmic reticulum. Afterward, the immature protein is transported to the Golgi apparatus, where it will associate with other myelin components, such as cholesterol, galactocerebrosides, and sulfatides [49], to form lipidic rafts [50,51] (Figure 3). Then, the lipidic rafts are sent toward the cellular membrane to form the myelin sheath [52]. In addition to its role of membrane anchoring, the fatty acylation of the N-terminal of PLP1 acts as a signal to target the delivery of PLP1 to newly formed myelin portions [53].

DM20 and PLP1 have the same topology [51], but DM20 does not have the part of the loop with two intracellular acetylation sites; this explains the conformational and steric differences observed between the two proteins [54,55]. DM20 and PLP1 interact together to form heterodimers [56]; however, their functional differences are not yet fully understood. It is known that PLP1 is necessary to maintain the integrity and the compaction of myelin sheaths [57], and it plays a role in axonal development and survival [58,59]. Their roles in the PNS are even more elusive, where the expression of DM20 exceeds that of PLP1. The amounts of PLP1 and DM20 are less than 0.01% of the total proteins of the PNS myelin [44]. While a border cannot be drawn between the physiological roles of PLP1 and DM20, the two proteins are not equivalent. For instance, DM20 cannot fully compensate for the loss of PLP1 even in isoform-specific knockout mice models [45,47] or in humans [29,34].

In addition to its structural role, PLP1 is suspected of playing a functional role as an ion or small molecule channel [60]. The idea that PLP1 might have more than a structural role was understood after it was known that the Plp1 gene was active long before the beginning of myelination [46,61]. In addition, Plp1 gene products have been detected in CNS non-myelinating cells, for instance, in olfactory ensheathing cells [62], brainstem neurons [63], cerebellum, and hippocampus neurons [64], and satellite cells [65]. Furthermore, neuronal PLP isoforms have been detected during human fetal development that is addressed as classical PLP proteins to the plasma membrane [66]. Finally, non-nervous tissues, such as the heart [67], the spleen [68], the fetal thymus [68], the thyroid, the trophoblasts, the spermatogonia, and the skin [69], have also been found to use Plp1 gene products. However, the specific roles of the proteins remain ununderstood.

## 5. Genetic Intricacy and Phenotypic Repercussions

### 5.1. Point Mutations: The Most Diverse Genetic Alterations

**Figure 4:**
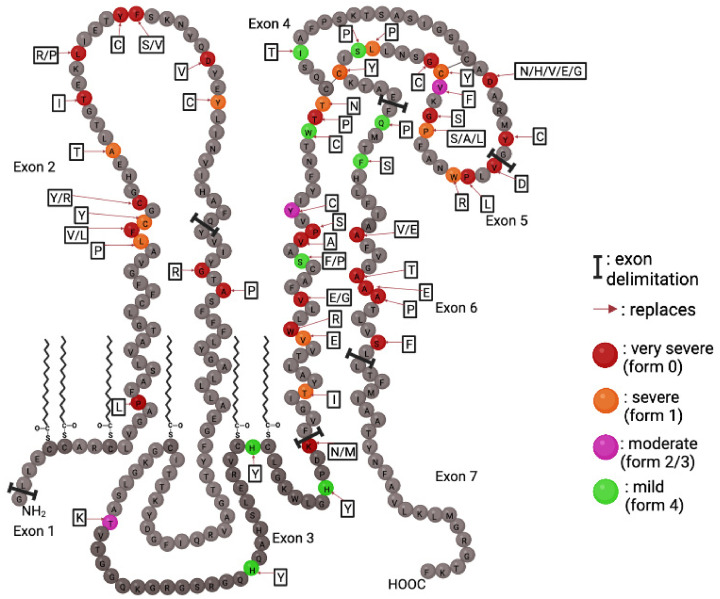
Point mutations of PLP1 and the severity of their repercussions. PLP1 point mutations are color coded to reflect the severity of the caused pathological phenotype. Mutations can occur throughout all seven exons. Amino acid changes are shown in adjacent boxes.

A wide variety of *PLP1* genetic alterations have been identified as the underlying causes of PMD and SPG2. Understanding their cellular and biochemical effects is essential to appreciate the pathogenesis of the diseases illustrated by a genotype–phenotype correlation. The effects of pathological modifications of PLP1 gene products are better understood than the physiological roles of the PLP1 and DM20 proteins. After over 50 years of study, the roles of PLP1 are still not elucidated, but the high conservation of its entire sequence between species suggests that any mutation would have detrimental effects. Missense amino acid substitutions caused by point mutations account for approximately 20% of the genetic alterations found in PMD patients. The clinical phenotypes resulting from point mutations vary from mild (SPG2) to severe (connatal PMD). Mutations can be found anywhere in the coding exons of the *PLP1* gene, and each mutation is usually specific to a family of patients [28]. Therefore, no common alleles or founder populations have been described for PMD. Mutations affecting the exon 3B, which encodes the 35 amino acid PLP1-specific region, cause an SPG2 phenotype, as DM20 is presumably intact but not PLP1 [25,70,71]. In the same way, truncating mutations (frameshifting and nonsense) cause a mild pathological phenotype [72] (Figure 4).

These mutations do not result in the production of deleterious proteins because of the degradation of mutant mRNA by the nonsense-mediated decay (NMD) pathway that detects and eliminates mRNA with premature stop codons [73]. Additionally, mutations in the N-terminal third of PLP1 tend to cause severe pathological phenotypes [24], and mutations in the second extracellular domain can cause the full range of phenotypes [74]. The pathological phenotype is determined by a loss of function of the physiological protein (moderate) and a gain of toxicity of the mutated protein (severe). Moreover, deep intronic deletions in intron 3 of *PLP1* cause a severe PMD phenotype by altering the splicing of the mRNA [75]. Consequently, the severity of the phenotypical repercussions of a mutation is determined by its effects on protein misfolding and three-dimensional configuration [74].

At a cellular level, the secretory pathway in oligodendrocytes is disrupted and related to PMD [76]. The mutant PLP1 accumulates inside the oligodendrocytes in the endoplasmic reticulum (ER), and cannot be delivered to the cell membrane [77]. The accumulation is caused by the stable binding of a PLP1 transmembrane domain with calnexin, an ER chaperon protein [78]. The prolonged binding of these two proteins prevents the proper functioning of the ER-associated protein degradation pathway to correctly eliminate the misfolded proteins [79].

**Figure 5:**
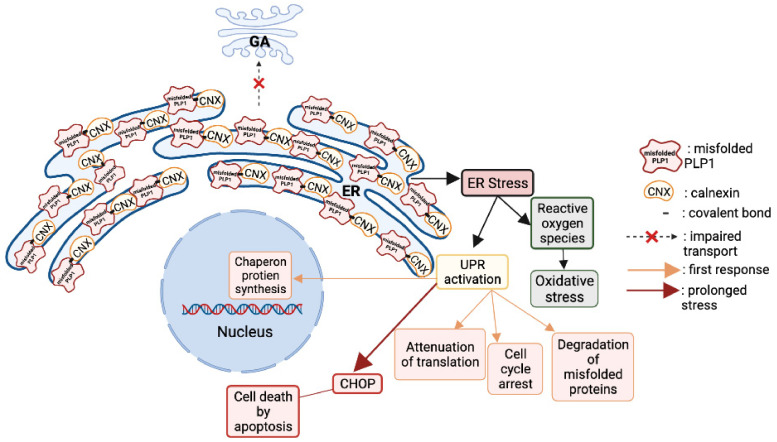
Consequences of PLP1 point mutations on the cellular physiology. The mutant PLP1 accumulates inside the oligodendrocytes in the endoplasmic reticulum (ER), and they cannot be delivered to the cell membrane. The accumulation is caused by the stable biding of a PLP1 transmembrane domain with calnexin, an ER chaperon protein. The prolonged binding of these two proteins prevents the proper functioning of the ER-associated protein degradation pathway to correctly eliminate the misfolded proteins. Moreover, the cytoplasmic accumulation of misfolded PLP1 and DM20 activates the unfolded protein response (UPR) pathway that aims to rescue the cell by stopping protein translation, degrading misfolded proteins, and increasing the production of chaperon proteins responsible of protein folding. When all the rescue mechanisms of the UPR are in vain, the ultimate response is to induce apoptosis by the activation of the CHOP pathway.

Moreover, the cytoplasmic accumulation of misfolded PLP1 and DM20 [79] activate the unfolded protein response (UPR) pathway that aims to rescue the cell by stopping protein translation, degrading misfolded proteins, and increasing the production of chaperon proteins responsible of protein folding [70]. When all the rescue mechanisms of the UPR are in vain, the ultimate response is to induce apoptosis by activation of the CHOP pathway (Figure 5).

UPR activation may be a common pathway in neurodegenerative disorders caused by the cytoplasmic accumulation of misfolded proteins and apoptotic cell death. Notably, Alzheimer’s disease, Parkinson’s disease, prion disease, Charcot–Marie–Tooth disease, and PMD possibly consists of UPR pathway activation involved in the development of a pathological phenotype [71,80] (Figure 5).

### 5.2. Gene Duplication: The Most Common Genetic Alteration

After the establishment of the fact that PMD is caused by a variety of point mutations, not all the cellular disease-causing mechanisms were elucidated. Many families with clinically declared PMD showed some dependence on the *PLP1* locus but did not have intragenic *PLP1* mutations [13]. A better understating of the pathological mechanisms has been performed by studying the dosages of the *PLP1*-gene-products [81] and the discovery of submicroscopic interstitial duplications of the X chromosome, including the PLP1 gene [82]. Genetic rearrangements resulting in duplications of segments containing the integrity of the PLP1 gene represent the most frequent (60 to 70%) underlying cause of PMD [14,83,84,85]. The interstitial Xq22.2 result in a transcriptionally active extra copy of the PLP1 gene that causes overexpression of the PLP1 and DM20 proteins [81,82]. In this case, dysmyelination is not caused by a misfolded protein but rather by a surplus of the normal/physiological protein. The dosage effect is a common pathological effect of a group of diseases caused by large genomic structural rearrangements [35,86]. PLP1 gene duplication is usually a tandem duplication involving large sections of neighboring genes [83,85]. The difference in breakpoints varies from one family to another [87], unlike other inherited genetic duplications where the duplicated segment is of a constant size, such as Charcot–Marie–Tooth 1A (CMT1A), because the duplication is caused by non-homologous recombination between flanking low copy repeats [88,89].

**Figure 6:**
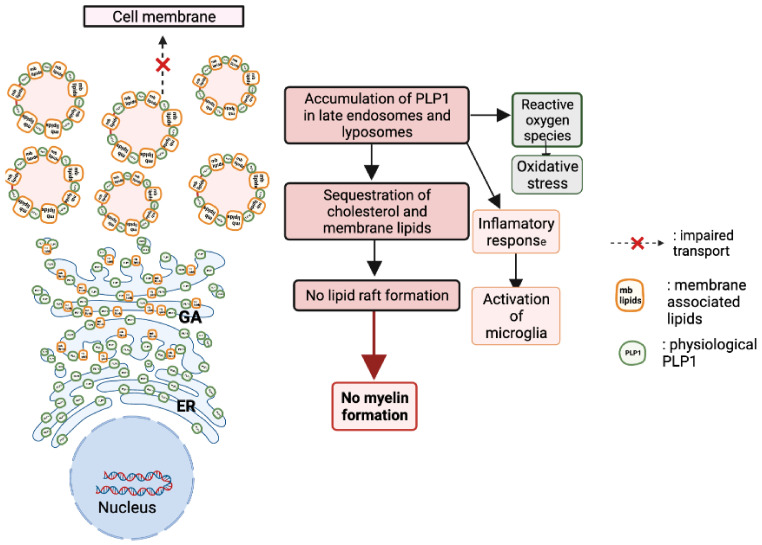
Consequences of PLP1 duplication on the cellular physiology. When overexpressed, PLP1 sequesters cholesterol and accumulates in late endosomes and lysosomes. Moreover, raft formation and membrane trafficking are crucial for myelin formation, and their disruption causes the loss of myelin. In addition to its cytotoxic effects, PLP1 protein overexpression leads to an inflammatory response with the activation of microglia in white and grey matter and the upregulation of cytokines and their receptors. It is probable that the microglial activation and the inflammatory response contribute to a certain extent to the pathology’s development and progression.

Moreover, patients with more than two copies of the *PLP1* gene (three to five copies) show a severe form of PMD with more severe symptoms [90]. When overexpressed, PLP1 sequesters cholesterol and accumulates in late endosomes and lysosomes. Moreover, the disruption of raft formation and membrane trafficking, which are crucial for myelin formation, cause the loss of myelin [50,91]. In addition to its cytotoxic effects, PLP1 gene overexpression leads to an inflammatory response with the activation of microglia in white and grey matter and the upregulation of cytokines and their receptors [92]. It is probable that the microglial activation and the inflammatory response contribute to a certain extent to the pathology’s development and progression (Figure 6).

In addition, mitochondrial defects have been linked to Plp1 overexpression. Plp1-overexpressing mice as well as human PLP1-duplicated fibroblasts had a severe malfunction of mitochondria paralleled with a severe ATP depletion [93] and abnormal interactions at the ER–mitochondria interface (MAM) [94].

### 5.3. Null Mutation: The Most Uncommon Genetic Alteration

A third pathological mechanism, other than the mutated *PLP1* and the overexpression of PLP1, is the absence of PLP1. The loss of function of PLP1 is caused by a complete deletion of the PLP1 gene [21,22,25] or by a point mutation at the beginning of the sequence that compromises translation or causes early termination of the translation [95,96]. Patients with null mutations have a milder PMD phenotype (form 3) or a complicated SPG2 form. These patients also suffer from mild peripheral neuropathy [95]. However, it is not specific for null-mutation patients—truncating mutations in the PLP1-specific region of exon 3 causes peripheral neuropathies and disrupts the PLP1:DM20 ratio [97]. This suggests that physiological PLP1 plays an important role in the peripheral nervous system and that DM20 is not able to compensate for its loss [98]. In contrast with other genetic alterations, deletions are extremely rare. Genomic mapping of the deleted sequences revealed that the deletions were significantly smaller than the duplications.

### 5.4. Mecanism of Neurological Manifestations in Heterozygotes Female Carriers

In most of the cases, female carriers are asymptomatic or paucisymptomatic, leading to an underestimation of their clinical state. Nevertheless, there are some exceptions, such as the original family studied by Pelizaeus and Merzbacher [7], where female carriers also exhibit some pathological symptoms. Considering the nature of the X-linked inheritance, female carriers have milder symptoms and later onset compared to the male patients in their families. However, considerable differences have been observed in female patients across several families with several investigators having observed inverse correlations between male and female patients. Some of the female carriers of mutations resulting in mild PMD forms in affected males have shown adolescent to adult onset with mild spastic diplegia and mild progressive leukodystrophy and dementia [99,100,101,102]. Conversely, in families with severely affected male PMD patients, the female carriers tend to show minimal clinical manifestations [32,80,103]. An unfavorable skewed X-inactivation could explain the contrast between male and female patients. Female carriers with random X-inactivation result in two distinct oligodendrocyte precursor cell lineages: one with the physiological *PLP1* gene and the other expressing the mutated allele. Severe *PLP1* mutations affect the maturation and differentiation of oligodendrocytes, leading to the apoptosis of the cells expressing the severely mutated protein. Consequently, the remaining mature oligodendrocytes are the oligodendrocytes expressing the physiological *PLP1*: a case of favorable skewing of X-inactivation and compensation. Whereas in female patients where the mutations of *PLP1* are less severe and are thus not lethal for the oligodendrocytes, the two oligodendrocyte cell lineages are viable and present in the CNS, leading to a mosaic oligodendrocyte population. Consequently, abnormal myelin is produced, causing manifestation of the disease in female patients.

Female carriers with *PLP1* duplications and not point mutations seem to have a favorable skewing of X-inactivation [104]. Thus, the development of PMD symptoms in female carriers depends on several factors, and one of them is severity of the mutation. Extrinsic factors that can affect myelination such as perinatal stress, an intrauterine infection, asphyxia, or hypoxia. They do not directly cause dysmyelination but rather increase susceptibility and delay the compensatory mechanisms. Some intrinsic factors may be deleterious polymorphisms in the other genes involved in the myelination pathway and neighboring-cell effect on the survival and competition between the *PLP1* alleles [105].

## 6. Animal Models

The progress in the understanding of PMD and the development of potential treatments could not be made without animal models for this disease. Among them, spontaneously occurring mutants for PLP1 have been of huge importance. One of the first PMD animal models was the *jimpy* mice described in 1954 [106]. The *jimpy* mice have abnormal CNS myelination due to mutations in the Plp1 gene. The deletion ranges from amino acid 208 to amino acid 232. However, the corresponding region is present in the *Plp1*-encoding gene. The *jimpy* mice have a single nucleotide mutation in their Plp1 gene; a base change from A to G in the 3′ acceptor splice site impairs the processing of the pre-mRNA [107]. This point mutation that alters the splicing of the mRNA [108] results in the elimination of the fifth exon [109] and thus the production of an abnormal PLP1. Both transcripts of this gene (PLP1 and DM20) are truncated in the 3′ end of their coding region. Compared to the wild-type sequence, the deletion is approximately 70 nucleotides long [110]. This deletion causes a frameshift in the open reading frame, causing an altered C-terminal for *jimpy Plp1* [10]. The *jimpy msd* mice have a single base substitution from C to T in exon 6 that would replace a valine by an alanine, causing a misfolded PLP1 [111]. The *jimpy 4j* also have a single mutation occurring in exon 2 that predicts the substitution of alanine at position 38 by a serine [112]. All mutations in *jimpy* mice occur at different locations, but all produce similar phenotypes with a severe deficiency of CNS myelin and oligodendrocytes, which die as soon as their fourth postnatal week. Moreover, spontaneous mutants with less severe phenotypes have been identified such as *rumpshaker* mice. These mice also have a point mutation in the Plp1 gene, causing the substitution of the isoleucine at position 186 by a threonine in a transmembrane domain of PLP1 [103]. Although the *rumpshaker* mice have defects in the levels of PLP1 and DM20 and clear evidence of hypomyelination, there are no effects on the oligodendrocyte viability and the lifespan of the animal [113]. The *rupmshaker* mice have been used as a model of SPG2 where the mice and the humans have the same mutation [114]. Myelin-deficient (*md*) rats also exist. The *md* rats have a point mutation (A to C) in exon 3, replacing the threonine in position 75 by a proline. This amino acid change occurs in the second transmembrane alpha-helix, causing a conformational modification prohibiting the protein from being integrated into the plasma membrane and causing the clinical manifestation of dysmyelination and premature death at 3 to 6 weeks of age [115]. Dogs have also been employed as models for PLP1 deficits. The “shaking pup” model has a single point mutation that substitutes a proline by a histidine near the first transmembrane domain of the protein. This mutation stops the maturation and differentiation of oligodendrocytes, and the dogs are characterized by severe hypomyelination, tremor, and early death [116]. Paralytic tremor rabbits have a point mutation in the PLP1 gene, a transversion from T to A in exon 2. This mutation causes the replacement of histidine (position 36) by a glutamine and the occurrence of dysmyelination and its clinical manifestations [117]. Recently, a novel non-human primate model of PMD has been described. Three neonatal rhesus macaques were reported with signs of hypomyelination, intention tremors, nystagmus, and motor dysfunctions. These monkeys have a missense mutation in exon 5 of the PLP1 gene [118].

In parallel to spontaneous mutants, transgenic models have been developed. A first line of transgenic mice overexpressing the Plp1 gene was produced by introducing a cosmid clone containing the entire mouse Plp1 gene. The mice with two more copies of the Plp1 gene had normal myelination until 3 weeks of age, but their state degraded afterward due to demyelination, whereas the mice having four extra copies of the Plp1 gene exhibited dysmyelination of the CNS. The overexpression of Plp1 prevented the oligodendrocytes from reaching their fully mature state, and the severity of this consequence correlated with the number of extra copies of the Plp1 gene. Furthermore, the abnormally high levels of PLP1 caused a swelling of the Golgi apparatus that disrupts cell trafficking and leads to apoptosis [119]. In parallel to the Japanese overexpressing mice, another model of *Plp1* has been developed at the Max Planck Institute of Goettingen. Two lines of normal mice expressing autosomal extra copies of the entire wild-type Plp1 gene were generated [120]. The first one, called line #66 and expressing 14 *Plp1* extra copies (+PlpTg^66/66^), developed severe hypomyelination, astrocytosis, seizures, and premature death (around 2 months of age). The second, called line #72 and expressing 6 *Plp1* extra copies (+PlpTg^72/72^), appeared behaviorally normal at 2 months of age but died prematurely 2–4 months later with severe seizures and convulsions due to important demyelination. These overexpressing mice further confirmed the importance of the Plp1 gene dosage regarding the terminal oligodendrocyte differentiation [120,121]. Latterly, the consequences of *Plp1* duplication were evaluated in the (Plp1dup) mouse model by introducing X chromosome duplication in the mouse genome that contains *Plp1* and five neighboring genes that are also commonly duplicated in PMD patients [122]. The effect of PLP1 overexpression was also modelized in rats. The PLP-transgenic (PLP-tg) rat was produced by microinjection of murine genomic *Plp1* sequences into the fertilized eggs of Lewis rats and modeled a severe form of connatal PMD [123,124]. In addition, animal models have been developed based on the third pathological mechanism affecting PLP1: null mutations and the absence of PLP1. The PLP1 deficient mice were employed to help unravel the physiological roles of the isoproteins PLP1 and DM20. These mice were produced by a mutation in exon 3 or the antisense insertion of a neocasette in exon 3 of the Plp1 gene. These modifications disrupt the expression of PLP1 and DM20, and the resulting mice do not express these proteins. The observed consequences are the disrupted apposition of the extracytoplasmic layers of the myelinating oligodendrocytes and the lack of dense lines. This disruption of the myelin sheath causes a reduction in the conduction velocities of CNS axons, impairment of neuromotor coordination, and behavioral changes [125]. Once again, Nave’s group developed a similar model that is more widely used by creating a true PLP/DM20 null allele, using a gene replacement vector that eliminates, by homologous recombination, the translation start site for PLP and DM20 [126]. In this model, despite the absence of both PLP and DM20, oligodendrocytes were still competent in assembling compacted myelin sheaths around the axons of all calibers. However, ultrastructurally, the electron-dense “intraperiod” lines in myelin remained condensed, correlating with its reduced physical stability. With time, abnormal compaction of myelin was observed to be associated with axonal swelling and degeneration, leading to the development of late (around 12 months) motor defects [59] mimicking the symptomatology of SPG2 patients [127]. Finally, while spontaneous mutants exhibited point mutations, and some of them were also generated in transgenic mice. Hence, mice carrying the deletion of a splicing enhancer in *Plp1* intron 3, causing a mild form of PMD and reducing PLP1-specific splicing in vitro, have been developed [34]. The main interest of all these animal models was to better understand and characterize the pathophysiological processes induced by the different mutations observed in PMD patients and to propose dedicated therapeutic targets [128] (Table 1).

## 7. Therapies

There is no definitive cure for PMD, and the treatments are mainly symptomatic and palliative. Patients suffering from connatal PMD with seizures are usually responsive to antiepileptic drugs (AED), with no specific AED used for the treatment of PMD. One of the major PMD symptoms is muscle spasticity. To help relieve patients, centrally acting skeletal muscle relaxants, such as baclofen, tizanidine, or diazepam, are administered. PMD patients with severe scoliosis are treated by surgery. For patients having difficulty feeding due to pharyngeal weakness, a gastrostomy is performed, and an enteral feeding is put in place [129]. Early neurophysiological diagnosis and physical rehabilitation are necessary to help improve the quality of life of PMD patients [130].

Research on the development of a treatment for PMD is still ongoing and is mainly supported by preclinical data. Different drugs able to reduce the toxicity of PLP1 overexpression have been identified. Lonaprisan, a drug from the progesterone receptor antagonist class, lowers the rates of toxic PLP1 levels caused by PLP1 overexpression in +PlpTg^72/72^ mice [131]. Curcumin has been shown to improve motor function and oligodendrocytes survival in the same mice model [132]. Dietary modifications have been tested on mice models of PMD. A diet rich in cholesterol can improve the life span of oligodendrocytes and helps in axonal survival. Dietary cholesterol supplementation does not cure PMD but rather helps relieve the oligodendrocytes from the cytoplasmic accumulation of PLP1 [133]. The ketogenic diet, which is high in fat and low in carbohydrates, helps to restore the oligodendrocytes, thus improving myelination in +PlpTg^72/72^ mice due to the ketone bodies that can cross the blood–brain barrier and that enhance lipid synthesis [134].

The feasibility of cell-based therapy has been tested in both mice and humans. Using a model of *Plp1* overexpression (+PlpTg^66/66^ mice), the remyelination potential of human oligodendrocyte progenitor cells (hOPCs) or human neural precursor cells (hNPCs) has been evaluated consequently regarding injection into the brain. While the onset of exogenous remyelination was earlier in hOPCs-grafted mice than in hNPC-grafted mice, extended lifespan occurred only in hNPCs-grafted animals [135]. In humans, a one-year open-label phase-1 study was undertaken to evaluate the safety and the remyelination potential of human central nervous system stem cells (HuCNS-SC) transplantation. Allogeneic HuCNS-SCs were surgically implanted into the frontal lobe of four males with a severe form of PMD. In three of the four subjects, durable cell engraftment and donor-derived myelin formation associated with modest gains in neurological function were observed [136]. The four years follow-up of these patients confirmed the good tolerance to this procedure and revealed persistent increased signal changes in the three patients [136].

Umbilical cord blood transplant has also been performed on two young PMD patients from an unrelated donor. It is stated that stem cells derived from the umbilical cord have the capacity to accelerate brain myelination [137]. The aim was to stabilize the state of the patients and to slow down progression of the disease [138].

The efficiency and feasibility of potent RNA-based treatments are being investigated. Antisense oligonucleotides (ASOs) have been beneficial to a mouse model of PMD overexpressing PLP [139]. However, this approach has many limitations. First, it is based on the complete suppression of PLP1, which is known to cause SPG2 and milder forms of PMD. Furthermore, the antisense oligonucleotides do not cross the blood–brain barrier and must be invasively administered by an intracerebroventricular (ICV) injection. Another trial based on micro-RNA (miRNA) was tested. A PLP1-targeting artificial miRNA cassette delivered by a scAAV vector was injected into *Plp1-*Tg mice in an attempt to decrease the levels of PLP1 [140]. The authors showed an improvement in survival and of neurological phenotypes.

## 8. Conclusions

Since the characterization of PMD over a century ago, several therapeutic approaches have been tested. However, no satisfactory treatments have been developed. Today, PMD is still an uncured disease with the quality of life of the patients deteriorating over time, and often causing premature death. Currently, the diagnostic tools and the overall knowledge allow for the accurate and early diagnosis of PMD. Patients receive palliative treatments to ease their pain, but an effective cure for PMD is urgently needed.

## Figures and Tables

**Table 1:** PMD animal models. The most routinely employed PMD models have been developed to study the mechanisms and pathological features of PLP1 alterations and to test treatments.

Animal Model	Species	Genetic Alteration	Protein Alteration	Pathological Features	References
*Jimpy*	*Mus musculus*	A > G 3′ end	Del. Aa 208 > 232	Dysmelination, gliosis, glial apoptosis	[110]
*Jimpy msd*	*Mus musculus*	C > T exon 6	Val > Ala	Dysmelination, gliosis, glial apoptosis	[111]
*Jimpy* 4*j*	*Mus musculus*	Exon 2 mutation	Ala 38 > Ser	Dysmelination, gliosis, glial apoptosis	[112]
*PlpTg* ^66/66^	*Mus musculus*	14 extra copies	Dose effect	hypomyelination, astrocytosis, seizures,	[120]
*PlpTg* ^72/72^	*Mus musculus*	6 extra copies	Dose effect	Seizures, convulsions, demyelination,	[121]
*Rumpshaker*	*Mus musculus*	Point mutation	Ile 186 > Thr	Hypomyelination	[103]
*PLP null*	*Mus musculus*	Neo cassette exon 3	-	Hypomyelination	[125]
*md rats*	*Rattus norvegicus*	A > C exon 3	The 75 > His	Dysmelination, gliosis, glial, apoptosis	[115]
*Shaking pups*	*C. lupus familiaris*	Point mutation	Pro > His	Dysmelination, glial activation apoptosis	[116]
*pt rabbits*	*O. cuniculs*	T > A exon 2	His 36 > Gln	Dysmyelination, gliosis	[117]
*RM PMD*	*Macaca mulata*	T 682 > C	Cys 228 > Arg	Dysmelination, glial activation, apoptosis	[118]

## Data Availability

Not applicable.
